# Coordinated proteome-scale remodeling underlies polyextremophilic survival in Antarctic cryo-hypersaline brines

**DOI:** 10.3389/fmicb.2026.1822442

**Published:** 2026-04-17

**Authors:** Shubham Pandey, Anjali Gupta, Ashwini Chauhan, Mohammad Ali Amoozegar, Ram Karan

**Affiliations:** 1 Department of Microbiology, University of Delhi South Campus, New Delhi, India; 2 Department of Microbiology, School of Biology, College of Science, University of Tehran, Tehran, Iran

**Keywords:** Antarctic Deep Lake, charge–hydrophobicity remodeling, cryo-hypersaline adaptation, *Halorubrum lacusprofundi*, Martian cryo-brine analog, proteome acidification, structural flexibility

## Abstract

**Introduction:**

Cryo-hypersaline brines combine sub-zero temperatures with near-saturated salinity, creating one of Earth's most extreme habitats. Antarctic Deep Lake provides a natural model for studying how proteins remain stable and functional under this dual stress and serves as a terrestrial analog for Martian cryo-brines.

**Methods:**

We performed a comparative proteome-scale analysis of *Halorubrum lacusprofundi*, the dominant haloarchaeon of Antarctic Deep Lake, to define the molecular basis of protein function under simultaneous cold and hypersaline stress. High-confidence structural prediction was integrated with genome-wide physicochemical profiling of more than 3,000 proteins and comparative analysis against mesophilic, psychrophilic, and halophilic reference organisms was performed.

**Results:**

The cryo-hypersaline proteome displayed pronounced acidic enrichment, lower isoelectric points, reduced hydrophobicity, and extensive surface charge redistribution, consistent with enhanced solubility under high ionic strength. However, flexibility profiling showed that this acidic, highly charged framework is not accompanied by uniform rigidification; instead, conformational dynamics were selectively preserved in functionally important regions. Substitution analysis further supported a layered adaptation strategy in which halophilic acidification is retained while cold-relevant mobility is superimposed on this background.

**Discussion:**

These results indicate that the defining feature of cryo-hypersaline adaptation is not any single exclusive structural trait, but the coordinated integration of halophilic solubility determinants with selective dynamic tuning for low-temperature function. Together, this work establishes a multi-layered adaptive framework for protein persistence in Antarctic cryo-brines and provides insight into molecular adaptation in polyextreme environments, including habitats relevant to Martian cryo-brines.

## 1 Introduction

Protein stability under extreme environmental conditions is constrained by fundamental physicochemical forces governing solvation, electrostatic interactions, and conformational dynamics. Elevated salinity increases ionic strength, disrupts hydrophobic packing, and enhances competition for hydration shells, thereby promoting protein aggregation and structural destabilization (Karan and Khare, 2011; Karan et al., 2011a,b; Kumar et al., 2012; Pandey et al., 2025a,b). In contrast, low temperature reduces molecular flexibility, decreases conformational entropy, and limits catalytic turnover rates. When these stresses coexist, as in cryo-hypersaline environments, proteins must maintain solubility in near-saturated brines while preserving sufficient backbone mobility to sustain enzymatic activity at sub-zero temperatures (Karan et al., 2012a,b, 2013, 2020; Laye et al., 2017). Because these adaptations can appear partially opposing when halophilic and psychrophilic traits are considered independently, an important unresolved question is whether cryo-hypersaline proteins display conflicting structural signatures or a scale-dependent integration of both. Although individual adaptations to either salinity or cold have been investigated (DasSarma et al., 2013; Karan and Khare, 2010; Renn et al., 2021; Sysoev et al., 2021), the integrated molecular strategies that enable simultaneous tolerance to both stresses remain insufficiently resolved.

Antarctic Deep Lake represents a permanently liquid, hypersaline ecosystem in which these combined constraints are sustained year-round. With salinity levels reaching 21–28% (w/v) and temperatures ranging from 11.5°C to −18°C, this system provides a natural framework to examine protein behavior under continuous cold and ionic stress (DasSarma, 2006; DasSarma et al., 2013; Karan et al., 2020; Laye and DasSarma, 2018; Williams et al., 2014). In addition to its terrestrial significance, Deep Lake serves as a geochemical analog for proposed saline brines on Mars, where low temperature and high salt concentrations may transiently coexist (Dundas et al., 2017; Stillman et al., 2014).

The haloarchaeon *Halorubrum lacusprofundi* dominates this environment and is widely used as a psychrohalophilic model organism (DasSarma et al., 2020). Enzyme-level investigations have demonstrated that individual proteins from this archaeon retain catalytic competence under cold and hypersaline conditions, supported by features such as acidic residue enrichment and surface charge redistribution (Karan et al., 2013, 2020; Laye et al., 2017). However, most previous studies have focused on isolated proteins, leaving unresolved whether these traits represent local adaptations or reflect coordinated restructuring across the entire proteome.

A key unresolved question is whether cryo-hypersaline survival arises from cumulative enzyme-specific adjustments or from systematic proteome-scale remodeling of physicochemical properties. Moreover, it remains unclear whether observed acidic enrichment and charge redistribution are actively selected structural features or indirect consequences of genomic nucleotide composition. Addressing these questions requires an integrated analysis combining structural modeling, compositional profiling, and directional substitution mapping across multiple functional systems.

In this study, we performed a comparative proteome-wide investigation of *H. lacusprofundi* alongside carefully selected environmental reference systems: the mesophile *Escherichia coli*, a well-characterized bacterial model with extensive structural and proteomic annotation; the psychrophile *Psychromonas ingrahamii*, adapted to permanently cold marine environments; and the halophile *Haloferax volcanii*, a genetically tractable archaeal model for hypersaline adaptation. These organisms represent distinct thermal and salinity optima, enabling resolution of adaptive features associated with single vs. combined environmental extremes. High-confidence structural predictions were integrated with analyses of residue composition, isoelectric point distribution, hydropathy, electrostatic surface potential, conformational flexibility, and amino acid substitution patterns across seven conserved enzyme families and more than 3,000 proteins per organism.

We tested the hypothesis that adaptation to cryo-hypersaline conditions is achieved through coordinated proteome-scale remodeling characterized by systematic acidification, hydrophobicity reduction, redistribution of charged residues, and selective tuning of conformational dynamics. By integrating enzyme-level and proteome-wide analyses, this work defines the molecular architecture underlying survival in Antarctic cryo-brines and clarifies the hierarchical mechanisms driving polyextremophilic adaptation.

## 2 Materials and methods

### 2.1 Sequence retrieval and structural modeling

A comparative dataset of homologous enzymes from four different microbial niches- the mesophile *Escherichia coli* (E. *coli*), the psychrophile *Psychromonas ingrahamii*, the halophile *Haloferax volcanii* and the psychrohalophile *Halorubrum lacusprofundi* was created in order to carefully investigate the molecular strategies of polyextremophilic adaptation (Table 1, Supplementary Table S1). Adenylate kinase (Adk), β-galactosidase (β-Gal), glycerol kinase (GlpK), histidinol-phosphate aminotransferase (HisC), DNA ligase (LigA), malate dehydrogenase (MDH), and signal recognition particle 54 (SRP54) are seven functionally distinct protein families that were chosen to symbolize essential biological functions spanning from core metabolism to replication and cell division (Table 2). Three-dimensional models for these essential enzymes were predicted using the SWISS-MODEL/AlphaFold2/ESMFold v1.0 model, utilizing the evolutionary scale language model (Bienert et al., 2017; Lin et al., 2023; Varadi et al., 2022). The processed structures were visualized and analyzed using the PyMOL Molecular Graphics System, Version 3.1.6.1. Prior to electrostatic mapping, all solvent molecules, heteroatoms, and ligands were computationally removed to isolate the proteinaceous contribution to the surface potential.

**Table 1:** Environmental and genomic characteristics of model organisms used for comparative analysis.

Organism	Adaptation	Temp. range (°C)	Salinity range %^*^	Natural habitat	Genome size (Mbp)	Average proteome pI
*Escherichia coli*	Mesophile	10 to 46	< 1	Mammalian gut (laboratory model)	4.64	6.90
*Psychromonas ingrahamii*	Psychrophile	−12 to 10	1–10	Polar marine and sea-ice environments	4.56	7.45
*Haloferax volcanii*	Halophile	30 to 50	10–23	Hypersaline lakes and salterns	4.01	5.08
*Halorubrum lacusprofundi*	Psychrohalophile	−18 to 12	21–28	Antarctic Deep Lake cryo-hypersaline brine	3.74	4.39

^*^(% w/v NaCl or equivalent).

**Table 2:** Core enzymes selected for comparative structural analysis across environmental gradients.

Enzyme	Functional significance	Structural relevance to this study
Adenylate kinase (Adk)	Energy homeostasis	Dynamic enzyme illustrating stability—plasticity balance
β-Galactosidase (β-Gal)	Carbohydrate metabolism	Large oligomeric structure sensitive to surface charge remodeling
DNA ligase (LigA)	DNA replication and repair	Tests stability of replication machinery under ionic stress
Glycerol kinase (GlpK)	Carbon utilization	Reflects metabolic adaptation under osmotic stress
Histidinol-phosphate aminotransferase (HisC)	Amino acid biosynthesis	Represents conserved housekeeping enzyme stability
Malate dehydrogenase (MDH)	Central metabolism (TCA cycle)	Conserved enzyme widely used for comparative structural studies
Signal recognition particle 54 (SRP54)	Protein targeting GTPase	Assesses conformational flexibility under dual stress

### 2.2 Electrostatic surface potential analysis

Surface charge distributions were calculated using PyMOL's Coulombic vacuum electrostatics approach. The AMBER 99 force field was used to give formal charges and atomic radii to the protein atoms. Electrostatic potentials were estimated in a vacuum (dielectric constant = 1.0) to emphasize the extremophilic surfaces' inherent charge distributions, independent of solvent screening effects. The electrostatic potential maps were standardized to a broad linear color ramp from −100 k_b_T/e (red, acidic/negative) to +100 k_b_T/e (blue, basic/positive) to fully depict the magnitude of the highly charged halophilic surfaces. Regions exhibiting near-zero potential were rendered in white. High-resolution figures were generated using the internal ray-tracer with shadows and depth-cueing disabled to ensure that charge distribution within deep active site clefts remained visible and quantifiable.

### 2.3 Conformational dynamics and flexibility simulation

To assess the flexibility of the target enzymes' structures, coarse-grained molecular simulations were carried out for each protein target's structure. The CABS-flex 2.0 server was used for coarse-grained molecular dynamics simulations (Kuriata et al., 2018). The homology models for *E. coli* (mesophile), *P. ingrihamii* (psychrophile), *H. volcanii* (halophile), and *H. lacusprofundi* (psychrohalophile) were used for simulations. The simulations were performed using default settings for the CABS-flex server algorithm. The algorithm settings used were protein rigidity equal to 1.0 and restraint weighting equal to 1.0. For visualization, representative structures were mapped and compared. These structures were superimposed onto each other. For proper visualization, comparative analysis of structures was done using the PyMOL software (The PyMOL Molecular Graphics System, Version 3.0 Schrödinger, LLC).

### 2.4 Physicochemical characterization of amino acid composition

The primary amino acid sequences of the selected homologs were analyzed to identify specific compositional biases associated with environmental adaptation. The percentage enrichment of acidic residues (aspartic acid and glutamic acid) was calculated to quantify the extent of surface acidification in halophiles. Additionally, the ratio of arginine (R) to lysine (K) was computed as a marker for psychrophilic adaptation. Global hydrophobicity was assessed by calculating the Grand Average of Hydropathicity (GRAVY) index using the Kyte-Doolittle scale to evaluate overall hydrophobic character and solvent interaction potential (Kastritis et al., 2013). DoGSiteScorer was used to detect potential binding pockets (Volkamer et al., 2010, 2012).

### 2.5 Amino acid substitution tracking and matrix generation

To determine the specific sequence-level substitution pathways driving the structural shifts observed in the targeted enzymes, pairwise amino acid substitution matrices were constructed. Global sequence alignments for the seven orthologous enzymes from *E. coli, P. ingrahamii, H. volcanii*, and *H. lacusprofundi* were generated using the Needleman-Wunsch algorithm (Align.PairwiseAligner, Biopython) with a BLOSUM62 matrix. Directional substitution frequencies between standard amino acids were tallied from the three environmental baselines to the polyextremophile *H. lacusprofundi*. Indels and strictly conserved residues were excluded to isolate targeted environmental adaptations. The resulting 20 x 20 matrices were visualized as 2D heatmaps utilizing a uniform colorimetric scale (vmax = 40) to ensure direct cross-panel comparability.

### 2.6 Genomic and proteomic mining

Full proteome sequences for *Escherichia coli* K-12 (TaxID: 83333), *Psychromonas ingrahamii* (TaxID: 357804), *Haloferax volcanii* DS2 (TaxID: 309800), and *Halorubrum lacusprofundi* (TaxID: 416348) were retrieved from UniProtKB (Bateman et al., 2025). Sequences shorter than 80 residues were excluded to minimize bias from peptide fragments. To assess whether proteome acidification reflects passive nucleotide composition or active structural selection, we performed a gene-level correlation analysis within each proteome. For each coding sequence, GC content was calculated as the proportion of guanine and cytosine among all standard nucleotides, and acidic residue frequency in the encoded protein was calculated as the percentage of aspartate and glutamate residues. Pearson correlation coefficients (*r*) between CDS GC content and encoded acidic residue frequency were computed separately for each organism.

### 2.7 Proteome-wide distribution and clustering analysis

Physicochemical properties such as isoelectric point (pI), GRAVY (Grand Average of Hydropathy), and amino acid composition were analyzed using the BioPython module “ProtParam” (Cock et al., 2009). Significance testing between the two groups was performed using the non-parametric Mann-Whitney U test as pI was not normally distributed. Biophysical stability was analyzed using the plot coordinates “Mean Net Charge” and “Mean Normalized Hydrophobicity” from Uversky plots to determine the intrinsic disorder possibility.

## 3 Result and discussion

This study integrates enzyme-level structural analysis with proteome-wide compositional profiling to examine molecular strategies enabling protein stability under combined cold and hypersaline stress. Comparative analysis was performed across four model organisms representing mesophilic, psychrophilic, halophilic and psychrohalophilic systems with distinct optimal growth conditions, inhabiting diverse niches (Table 1). Representative enzymes (Table 2), spanning genome maintenance, metabolism, protein targeting, and energy homeostasis were selected to ensure functional breadth (Cantarel et al., 2009; de Lorenzo et al., 2024; Dzeja and Terzic, 2009; Janda et al., 2010; Kulis-Horn et al., 2014; Williamson and Leiros, 2020; Zwaig et al., 1970). The rationale for selection was primarily threefold: first, these enzymes encompass a wide range of molecular weights and oligomeric states, allowing for the assessment of adaptation strategies across varying structural complexities; second, proteins like DNA Ligase are essential for cell survival and division, making their stability under stress non-negotiable; and third, metabolic markers like malate dehydrogenase (MDH) and adenylate kinase (Adk) serve as standard models for analysis of catalytic efficiency in extreme conditions. The consistent adaptive trends observed across these diverse systems indicate that psychrohalophilic survival reflects coordinated proteome-scale remodeling rather than isolated enzyme-specific changes (Goldenberg-Barbosa et al., 2025; Karan et al., 2020).

All enzymes were analyzed across mesophilic, psychrophilic, halophilic, and psychrohalophilic systems to evaluate structural remodeling, electrostatic adaptation, and conformational dynamics under combined temperature and salinity stress.

Comparative structural and physicochemical analyses revealed hallmark features of halophilic adaptation in *Halorubrum lacusprofundi*, superimposed in a cold environment. These include pronounced surface electronegativity, enrichment of acidic residues, reduced hydrophobicity, globally lowered isoelectric points and preferential substitution of certain amino acids in the proteins. Together, these characteristics define an integrated strategy that enhances solubility and structural persistence under sustained high ionic strength.

The complete enzyme-wise physicochemical parameters are provided in Supplementary Tables S2. Across the seven conserved enzymes examined, a consistent physicochemical shift was observed along the environmental gradient. When averaged across enzyme classes, mesophilic and psychrophilic systems maintained relatively neutral pI values and balanced charge–hydrophobic profiles, whereas halophilic and psychrohalophilic systems displayed a marked reduction in mean pI, elevated acidic residue enrichment, increased arginine-to-lysine ratios, and reduced hydrophobicity. These coordinated shifts indicate that adaptation to hypersaline conditions is not restricted to isolated enzymes but reflects a systematic remodeling of physicochemical properties at the cellular level.

### 3.1 Surface acidification and electrostatic stabilization of enzymes

**Figure 1:**
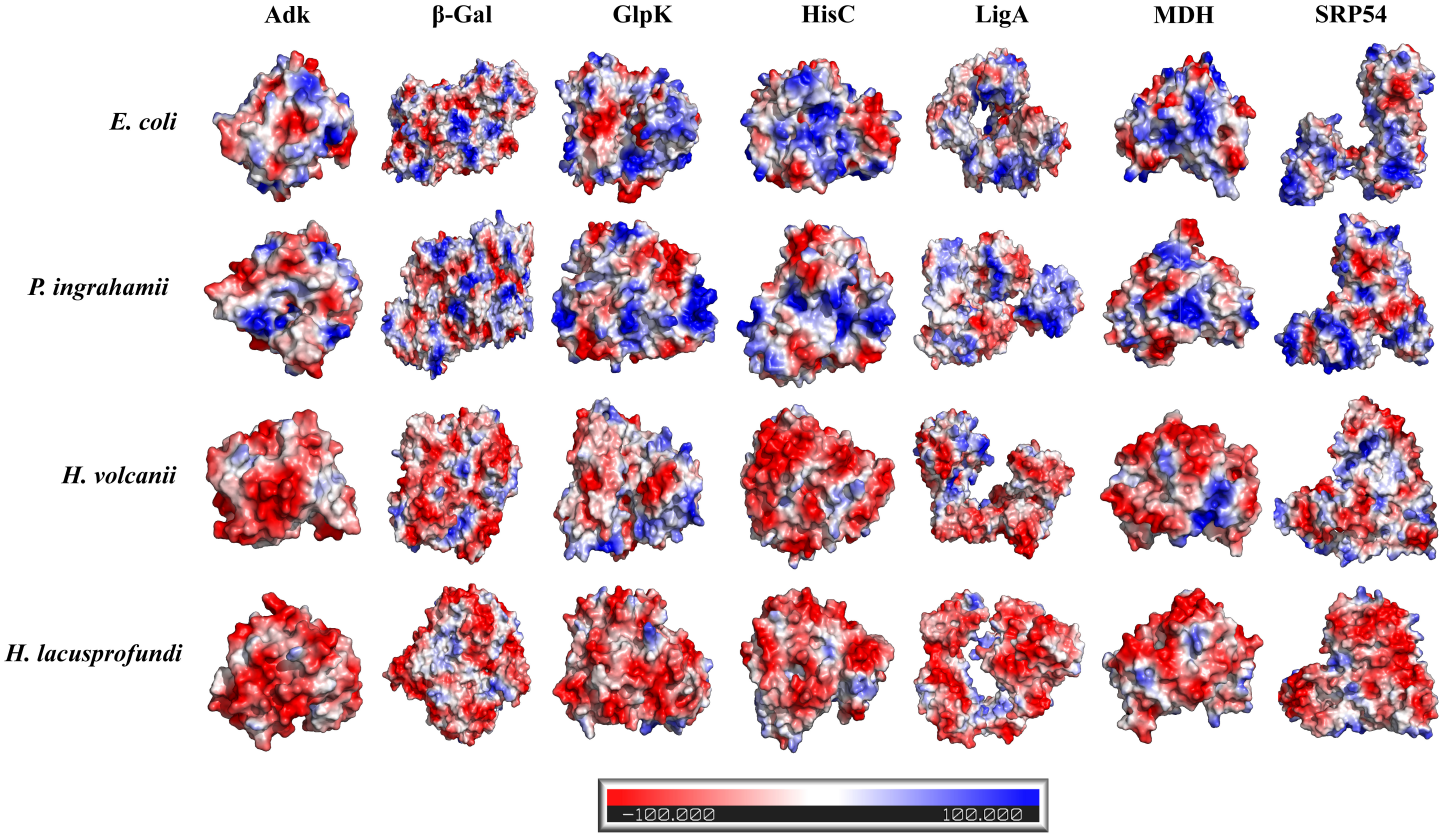
Surface electrostatic maps of adenylate kinase (Adk), β-galactosidase (β-Gal), DNA ligase (LigA), glycerol kinase (GlpK), histidinol-phosphate aminotransferase (HisC), malate dehydrogenase (MDH) and signal recognition particle 54 (SRP54) are shown for *Escherichia coli* (mesophile), *Psychromonas ingrahamii* (psychrophile), *Haloferax volcanii* (halophile), and *Halorubrum lacusprofundi* (psychrohalophile). Electrostatic potentials were calculated from predicted structures and mapped onto the molecular surface. Blue indicates positive electrostatic potential and red indicates negative electrostatic potential.

To examine surface electrostatic properties across environmental systems, electrostatic potentials were calculated for all modeled enzymes using the vacuum electrostatics module in PyMOL. Comparative visualization revealed consistent differences in surface charge distribution among the four organisms (Figure 1).

Proteins from the mesophilic reference system, *E. coli*, exhibited a heterogeneous surface pattern characterized by alternating regions of positive and negative charge. A similar patch-like distribution was observed in the psychrophilic organism *P. ingrahamii*, although the overall surface appeared less densely charged. In both systems, discrete electropositive regions were prominent, reflecting the presence of surface-exposed basic residues.

In contrast, enzymes from the halophilic system *H. volcanii* and the psychrohalophilic system *H. lacusprofundi* displayed a markedly different pattern. Their surfaces were dominated by continuous electronegative potential, due to enrichment of aspartic and glutamic acid residues forming extended acidic envelopes across large regions of the protein surface. This shift toward negative surface charge was consistent across all enzymes examined. Notably, the electronegative surface character was most pronounced in the psychrohalophilic system. In hypersaline environments, salt cations associate with negatively charged surfaces, promoting hydration and preventing aggregation. The predominance of acidic residues in these systems reduces surface hydrophobic exposure and is a recognized structural feature of halophilic adaptation (Herrero-Alfonso et al., 2024; Tadeo et al., 2009; Vogler et al., 2020).

In non-halophilic systems, elevated salt concentrations can promote dehydration and exposure of hydrophobic regions, leading to instability. By contrast, acidic enrichment in halophilic proteins supports retention of hydration shells even in near-saturated brines (Oren, 2013; Paul et al., 2008). These observations support the hypothesis that electrostatic remodeling represents a primary mechanism for protein stabilization in cryo-hypersaline environments. The persistence of this acidic architecture in the psychrohalophilic system suggests that salinity-associated electrostatic remodeling provides the dominant proteome-wide scaffold in Antarctic Deep Lake conditions, upon which additional cold-relevant adjustments are superimposed (Herrero-Alfonso et al., 2024; Vogler et al., 2020).

In classical psychrophilic proteins, cold adaptation is often associated with reduced rigidifying interactions and less extensive surface charge enrichment, helping preserve flexibility without requiring the strongly acidic surfaces typical of halophiles. In our dataset, this contrast was reflected by the psychrophilic reference *P. ingrahamii*, which displayed a more patch-like and less densely charged surface pattern, whereas *H. lacusprofundi* retained the continuous electronegative surface architecture characteristic of halophilic proteins. This indicates that, under combined cold and hypersaline stress, charge redistribution in *H. lacusprofundi* is shaped primarily by the demands of salt-dependent solubility and hydration rather than by the globally reduced-charge strategy often associated with psychrophilic conditions. Cold adaptation therefore appears to be accommodated mainly through selective tuning of flexibility on top of this halophilic electrostatic scaffold (DasSarma et al., 2013), reflecting the layered adaptive strategy for survival under such dual extremes.

### 3.2 Comparative RMSF-based flexibility profiling of representative enzymes

**Figure 2:**
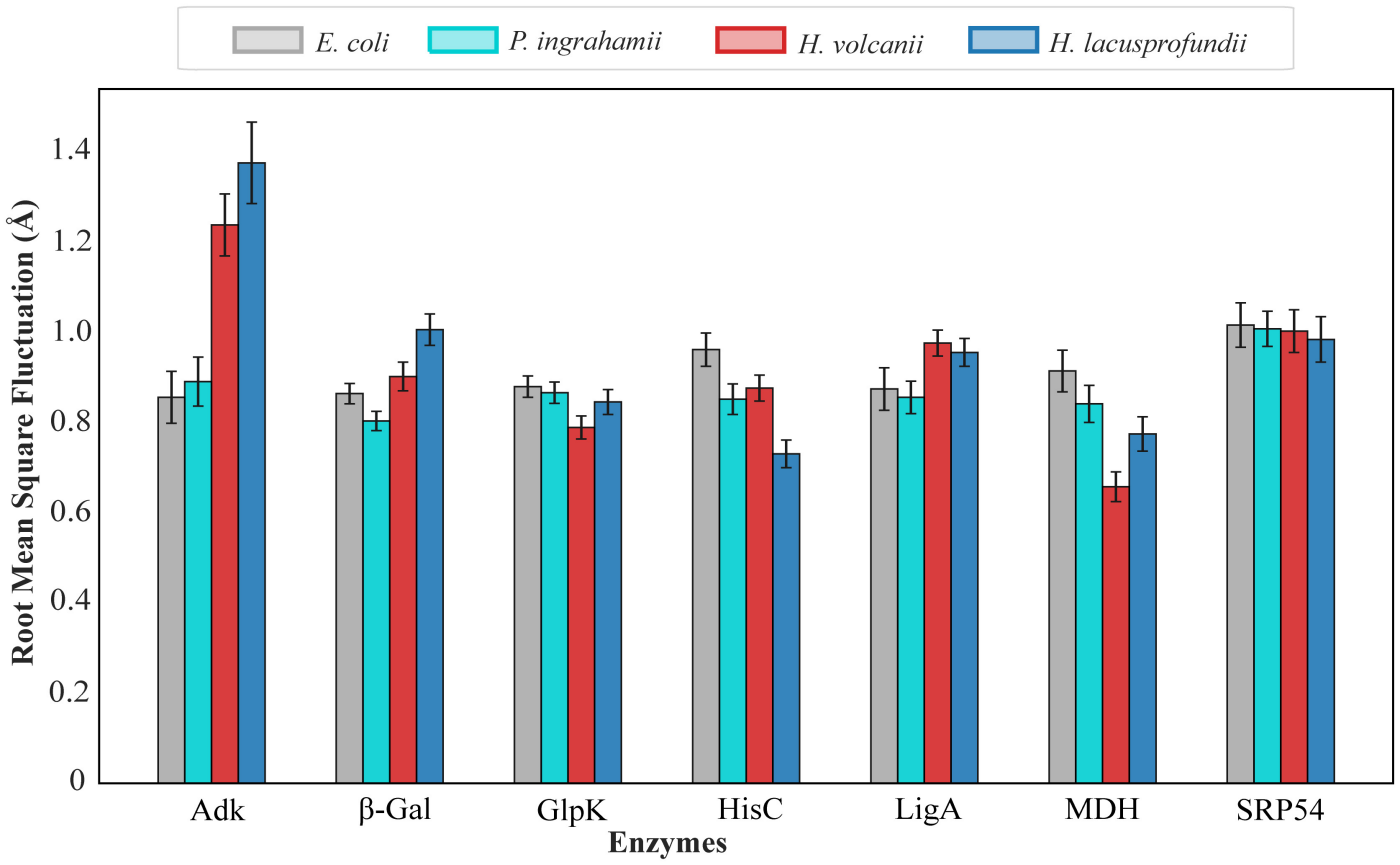
*Comparative conformational flexibility profiles of representative enzymes across environmental systems*. Average root mean square fluctuation (RMSF) values were calculated for adenylate kinase (Adk), β-galactosidase (β-Gal), DNA ligase (LigA), glycerol kinase (GlpK), histidinol-phosphate aminotransferase (HisC), malate dehydrogenase (MDH) and signal recognition particle 54 (SRP54) from *Escherichia coli* (mesophile), *Psychromonas ingrahamii* (psychrophile), *Haloferax volcanii* (halophile), and *Halorubrum lacusprofundi* (psychrohalophile). RMSF values represent residue-level fluctuations derived from structural models and are plotted as average values for each enzyme.

To assess conformational dynamics across environmental systems, residue-level flexibility was evaluated using root mean square fluctuation (RMSF) analysis (Figure 2). The resulting profiles revealed enzyme-specific modulation of flexibility rather than a uniform trend across organisms.

For glycerol kinase (GlpK), histidinol-phosphate aminotransferase (HisC), malate dehydrogenase (MDH) and signal recognition particle 54 (SRP54), higher average RMSF values were observed in the mesophilic (*E. coli*) and psychrophilic (*P. ingrahamii*) systems relative to their halophilic counterparts. This pattern is consistent with the established role of increased conformational flexibility in supporting catalytic efficiency at low temperatures (Xu et al., 2024).

In contrast, certain enzymes retained localized mobility despite global stabilization. Adenylate kinase and DNA ligase require domain movements during catalysis, and these dynamic regions appear selectively preserved (Laye et al., 2017; Vogler et al., 2020). In β-galactosidase, surface charge density may introduce controlled domain repulsion that prevents aggregation while maintaining sufficient mobility and activity (DasSarma et al., 2013; Karan et al., 2020).

Taken together, these results indicate that adaptation under combined cold and saline stress does not involve simple enhancement or reduction of flexibility. Instead, conformational dynamics appear to be redistributed in an enzyme-dependent manner, reflecting a balance between electrostatic stabilization and functional mobility. Thus, the psychrohalophilic response does not rely on globally weakening charge-based stabilization, but on retaining a halophilic charge architecture while redistributing mobility in functionally important regions.

Although kinetic parameters were not measured in the present study, the observed flexibility patterns have clear mechanistic implications. In cold-active enzymes, selective mobility in catalytic loops and surrounding regions is often linked to maintenance of turnover at low temperatures, sometimes with accompanying trade-offs in substrate affinity. In *H. lacusprofundi*, prior kinetic analysis of the polyextremophilic β-galactosidase demonstrated that subtle residue substitutions can alter catalytic efficiency at low temperature through temperature-dependent effects on both Km and kcat, with these changes traceable to perturbations in charge, hydrogen bonding, and packing around functionally important regions (Laye et al., 2017). Our results are therefore consistent with a model in which cryo-hypersaline adaptation preserves a halophilic solubility scaffold while selectively tuning local dynamics to support catalysis under sub-zero conditions.

### 3.3 Genomic and proteomic convergence under cryo-hypersaline conditions

**Figure 3:**
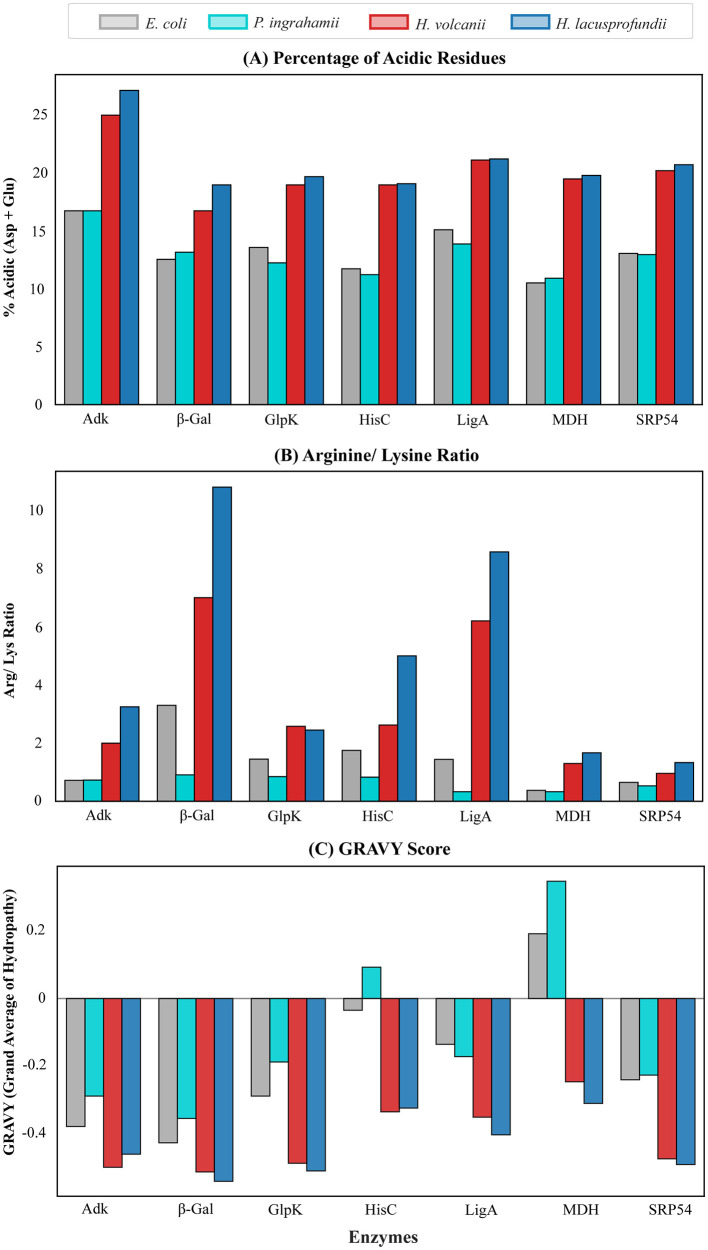
*Amino acid compositional features of representative enzymes across environmental systems*. **(A)** Percentage of acidic residues (aspartic acid and glutamic acid), **(B)** Arginine/ Lysine ratio, and **(C)** Percentage hydrophobicity of adenylate kinase (Adk), β-galactosidase (β-Gal), DNA ligase (LigA), glycerol kinase (GlpK), histidinol-phosphate aminotransferase (HisC), malate dehydrogenase (MDH) and signal recognition particle 54 (SRP54) from *Escherichia coli* (mesophile), *Psychromonas ingrahamii* (psychrophile), *Haloferax volcanii* (halophile), and *Halorubrum lacusprofundi* (psychrohalophile).

Amino acid composition analysis revealed consistent compositional shifts in enzymes from halophilic and psychrohalophilic systems relative to mesophilic and psychrophilic counterparts (Figure 3). Across all enzymes examined, *H. lacusprofundi* and *H. volcanii* homologs displayed elevated proportions of acidic residues (aspartic acid and glutamic acid), frequently exceeding 15% of total amino acid content. In contrast, enzymes from *P. ingrahamii* and *E. coli* exhibited comparatively lower acidic residue content.

This enrichment of acidic residues is a well-established feature of halophilic proteins and contributes to increased surface electronegativity, enhanced hydration, and improved solubility under high ionic strength conditions (Figure 3A; Grötzinger et al., 2018).

Consistent with this trend, halophilic and psychrohalophilic enzymes also exhibited higher arginine-to-lysine ratios relative to mesophilic and psychrophilic homologs (Figure 3B). Arginine residues, with their guanidinium side chains, are capable of forming more stable electrostatic and hydrogen-bonding interactions than lysine, potentially contributing to structural stabilization in hypersaline environments.

Hydrophobicity profiling further revealed a reduction in overall hydrophobic residue content in *H. lacusprofundi* and *H. volcanii* enzymes compared with *P. ingrahamii* and *E. coli* (Figure 3C). Lower hydrophobicity reduces aggregation propensity and favors solvent exposure, consistent with the requirement for high solubility in salt-saturated brines.

Similarly, the proteome-wide amino acid composition analysis revealed an increased percentage of small amino acid residues (19.68%), including glycine and alanine, which confer improved flexibility to the protein in comparison to the mesophilic host proteins (16.87%). Additionally, a reduced number of aromatic amino acids (phenylalanine, tyrosine, and tryptophan) was demonstrated by the psychrohalophilic proteins (7.03%) highlighting the halophilic trend of reduction in hydrophobicity as well as the reduced rigidity due to lesser bulky amino acid content (Supplementary Table S3).

The apparent contradiction between halophilic stabilization and psychrophilic flexibility is therefore resolved in *H. lacusprofundi* through a scale-dependent adaptive architecture, in which proteome-wide acidic solubility determinants are retained while cold-relevant mobility is selectively tuned at functionally important regions.

A related implication is that this tuning is likely to be regionally partitioned within multidomain enzymes. In polyextremophilic systems, catalytic cores may remain strongly conserved to preserve substrate recognition and reaction chemistry, whereas adaptive changes are more likely to accumulate in peripheral domains, surface loops, interdomain interfaces, or regions functionally coupled to the active site. This interpretation is consistent with earlier structural studies of *H. lacusprofundi* β-galactosidase, in which catalytic-site architecture was preserved while flexible loop regions and residue-level remodeling outside the catalytic core contributed to low-temperature function on a halophilic background (Karan et al., 2020; Laye et al., 2017). Thus, adaptation to combined cold and saline stress may be distributed according to structural context rather than uniformly across the entire protein.

### 3.4 Amino acid substitution biases underlying polyextremophilic adaptation

**Figure 4:**
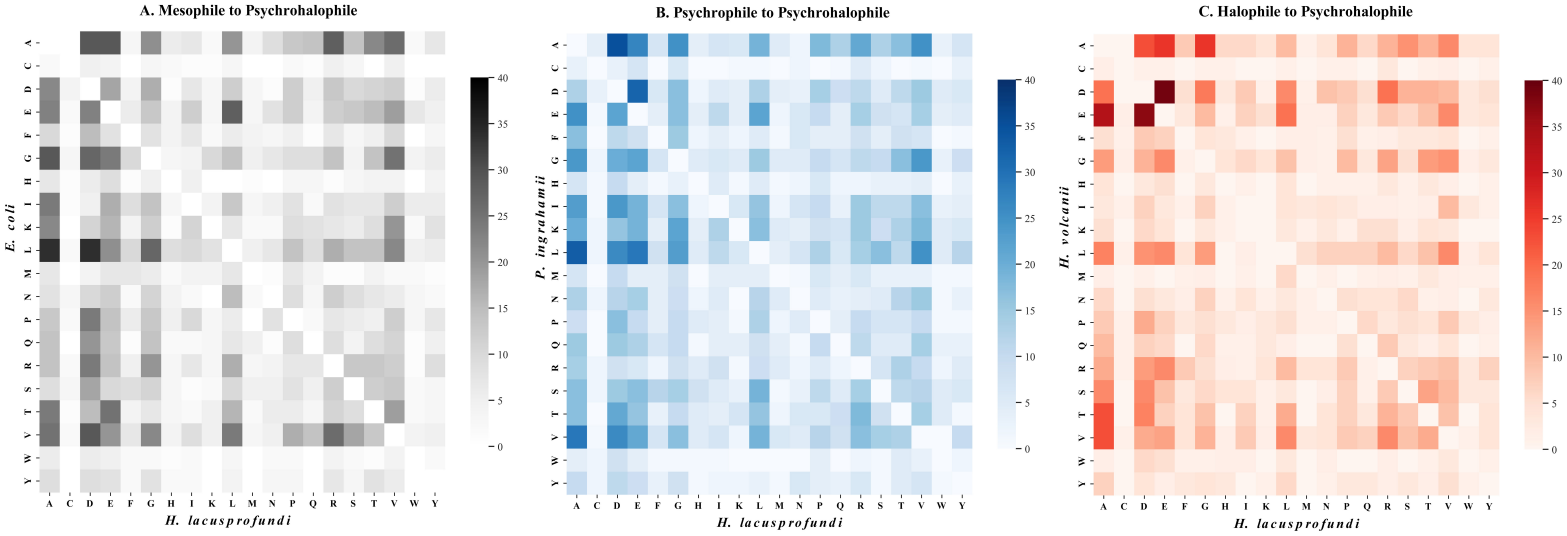
Amino acid substitution matrices defining the psychrohalophilic adaptation of *H. lacusprofundi*. Pairwise mutational frequencies across seven core enzymes, standardized to a maximum of 40 substitutions per heatmap. **(A)** Mesophile (*E. coli*) to psychrohalophile (*H. lacusprofundi*) transition **(B)** Psychrophile (*P. ingrahamii*) to psychrohalophile (*H. lacusprofundi*) comparison **(C)** Halophile (*H. volcanii*) to psychrohalophile (*H. lacusprofundi*) adaptation.

While proteome-wide analysis demonstrates systematic compositional remodeling in *H. lacusprofundi*, pairwise substitution profiling across seven conserved enzymes provides residue-level insight into the molecular shifts associated with combined cold and hypersaline adaptation (Figure 4).

Comparison between mesophilic and psychrophilic systems and the psychrohalophilic model (Figures 4A, B) reveal a clear directional bias toward acidic residue enrichment. Non-polar aliphatic residues, including alanine, leucine, and valine, frequently transitioned to aspartic acid and glutamic acid in the *H. lacusprofundi* homologs. For example, in the *E. coli* baseline, leucine-to-aspartic acid substitutions were observed 34 times across the enzyme set, while alanine was replaced by aspartic acid or glutamic acid more than 50 times (Supplementary Table S4). These substitutions collectively contribute to the pronounced acidic surface enrichment characteristic of halophilic adaptation (Karan et al., 2020).

In parallel, basic residues, particularly lysine, exhibited higher replacement frequencies relative to arginine, consistent with the elevated R/K ratios observed in the psychrohalophilic system. This pattern supports the interpretation that electrostatic stabilization under hypersaline conditions is achieved not only through acidic enrichment but also through selective redistribution of charged residues (DasSarma et al., 2013). The resulting acidic envelope reinforces solvation and charge screening in high ionic strength environments, in agreement with the electrostatic surface profiles described earlier (Figure 1).

The halophile-to-psychrohalophile comparison (Figure 4C) provides additional resolution into polyextremophilic adaptation. Because *H. volcanii* already exhibits extensive surface acidification, the substitution landscape between halophile and psychrohalophile is comparatively restrained in terms of charge remodeling. Instead, the predominant shifts involve residues associated with structural rigidity.

This pattern highlights a hierarchical adaptation strategy. In the transition from mesophile to psychrohalophile, acidification dominates. In contrast, when transitioning from an already halophilic background, adaptation appears to overlay psychrophilic flexibility onto a pre-existing acidic framework.

Together, these substitution biases indicate that polyextremophily in *H. lacusprofundi* reflects layered molecular remodeling rather than a single adaptive axis.

### 3.5 Proteome-scale compositional reprogramming

**Figure 5:**
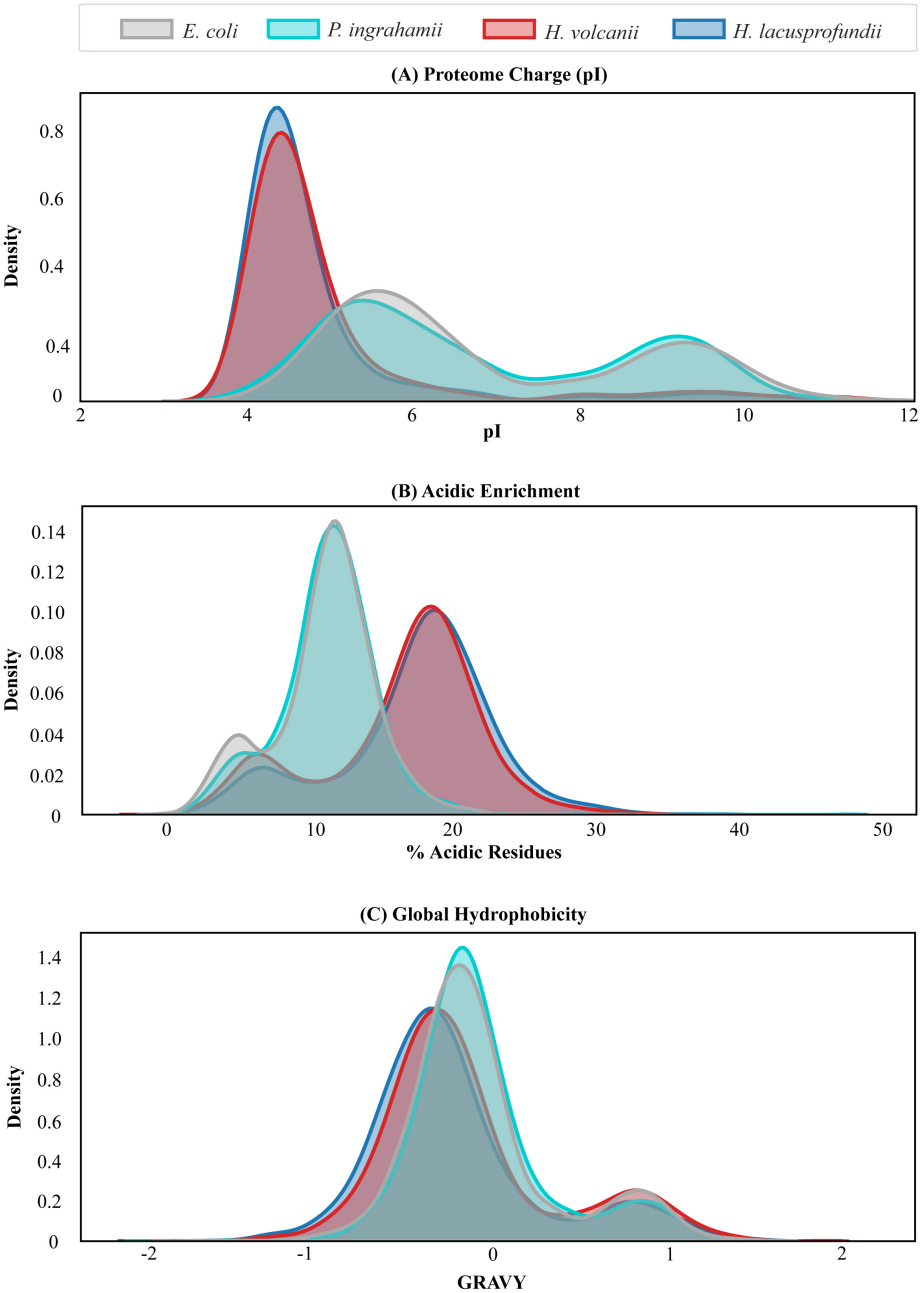
*Proteome-wide compositional signatures across environmental systems* **(A)** Distribution of isoelectric point (pI), **(B)** Proteome-wide enrichment of acidic residue, and **(C)** Global hydrophobicity (GRAVY) distribution. Data represent complete predicted proteomes of *Escherichia coli, Psychromonas ingrahamii, Haloferax volcanii*, and *Halorubrum lacusprofundi*.

To determine whether these compositional trends extend beyond selected enzymes, we performed proteome-wide analysis across more than 3,000 proteins from each organism (Figure 5).

In *E. coli* and *P. ingrahamii*, the distribution of isoelectric points (pI) displayed a bimodal pattern, with peaks centered around mildly acidic and mildly basic values, reflecting balanced representation of acidic and basic proteins. The global mean and median pI values were consistent with typical bacterial proteomes.

In contrast, the proteomes of *H. lacusprofundi* and *H. volcanii* exhibited a pronounced shift toward acidic pI values, with a dominant peak near pI ~4.5 (Figure 5A). This collapse toward a single acidic distribution reflects large-scale enrichment of acidic residues across the proteome (Oren, 2013; Tokmakov et al., 2021).

Consistently, acidic residue percentages across halophilic and psychrohalophilic proteomes were elevated relative to mesophilic and psychrophilic systems (Figure 5B), supporting charge-screening mechanisms in which environmental cations mitigate electrostatic repulsion between negatively charged residues (Akal et al., 2019; Gunde-Cimerman et al., 2018; Paul et al., 2008). The majority of proteins in the halophilic systems clustered within a higher acidic residue range compared with the narrower distribution observed in *E. coli*.

Global hydropathy analysis using GRAVY scores further supported this trend. Proteomes of *H. lacusprofundi* and *H. volcanii* exhibited more negative average GRAVY values, indicative of increased hydrophilicity relative to mesophilic and psychrophilic counterparts (Figure 5C), enhancing solubility in low-water, high-salt environments (Herrero-Alfonso et al., 2024; Karan et al., 2012a; Ortega-Quintanilla and Millet, 2025). Because hydrophobic core strength is relatively reduced in these systems, halophilic proteins rely more heavily on electrostatic interactions and salt-bridge networks for stabilization (Nayek et al., 2014; Zorgani et al., 2014). In contrast, mesophilic and psychrophilic proteins depend primarily on hydrophobic packing to maintain compact cores.

Together, these proteome-wide shifts demonstrate convergence toward acidic, hydrophilic protein architectures under sustained high-salt conditions. In the psychrohalophilic system, these compositional features coexist with cold-adaptive structural traits, indicating that salinity-oriented proteome-scale remodeling provides the dominant adaptive scaffold, upon which cold-relevant functional tuning is superimposed.

### 3.6 Biophysical positioning of proteomes in charge—hydrophobicity space

**Figure 6:**
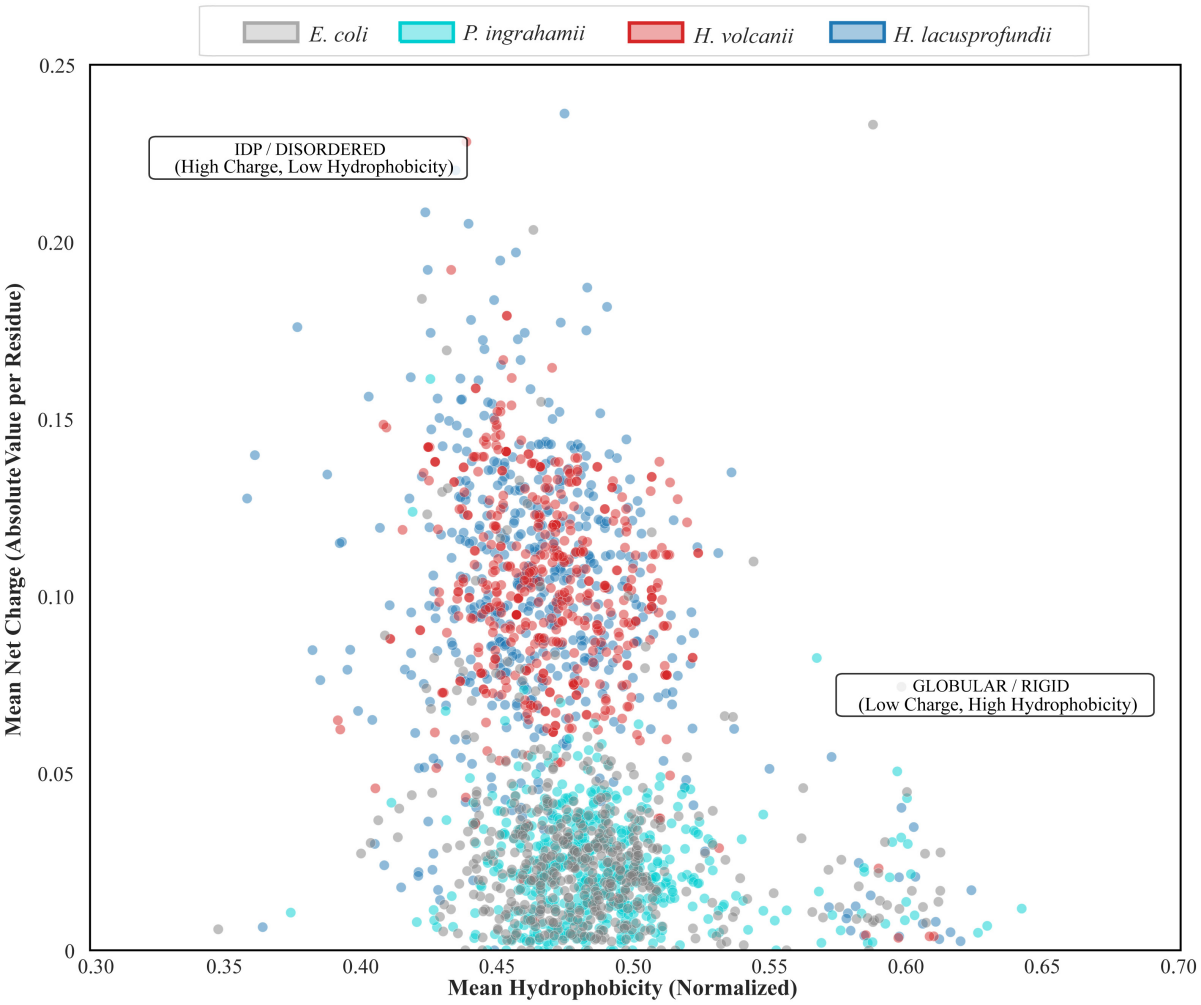
Uversky plot showing mean net charge vs. mean hydrophobicity for proteins from *Escherichia coli* (mesophile), *Psychromonas ingrahamii* (psychrophile), *Haloferax volcanii* (halophile), and *Halorubrum lacusprofundi* (psychrohalophile). The upper-left region corresponds to high charge and low hydrophobicity, while the lower-right region corresponds to low charge and higher hydrophobicity.

To integrate compositional trends with global biophysical behavior, proteomes were projected onto charge—hydrophobicity space using Uversky coordinates (Figure 6). This framework further contextualized global folding behavior (Zorgani et al., 2014).

Proteomes of *E. coli* and *P. ingrahamii* were distributed predominantly within the region corresponding to moderate net charge and relatively higher hydrophobicity. This positioning is consistent with compact, globular protein architectures typical of mesophilic and psychrophilic systems.

In contrast, proteomes of *H. volcanii* and *H. lacusprofundi* shifted toward higher net charge and reduced hydrophobicity. This displacement reflects enrichment of acidic residues and reduced hydrophobic core content observed in previous analyses. Although these proteomes approach regions associated with increased disorder in the Uversky framework, most proteins remain within the folded domain, indicating that intrinsic disorder is not the dominant adaptation.

The elevated net charge likely enhances electrostatic repulsion between protein molecules, reducing aggregation under high ionic strength conditions. Concurrently, lower hydrophobicity may limit unfavorable solvent exposure in saturated brines. Rather than indicating global disorder, this positioning suggests a redistribution of biophysical parameters to occupy a marginal stability regime characterized by elevated surface charge balanced by ionic shielding. Environmental salt mitigates destabilizing electrostatic repulsion while maintaining hydration (DasSarma et al., 2013; Laye et al., 2017). This balance allows structural plasticity under cold conditions without compromising overall folding integrity.

Together, the Uversky analysis supports a dual-adaptation model involving coordinated tuning of charge and hydrophobicity at the proteome scale, complementing enzyme-level structural remodeling observed in previous sections. These biophysical shifts further suggest that, in *H. lacusprofundi*, the primary proteome-wide response is shaped by hypersaline constraints, whereas cold adaptation is accommodated through selective tuning of local dynamics rather than an independent global compositional shift.

### 3.7 Proteome acidification reflects active structural selection and is largely decoupled from genomic GC bias

**Figure 7:**
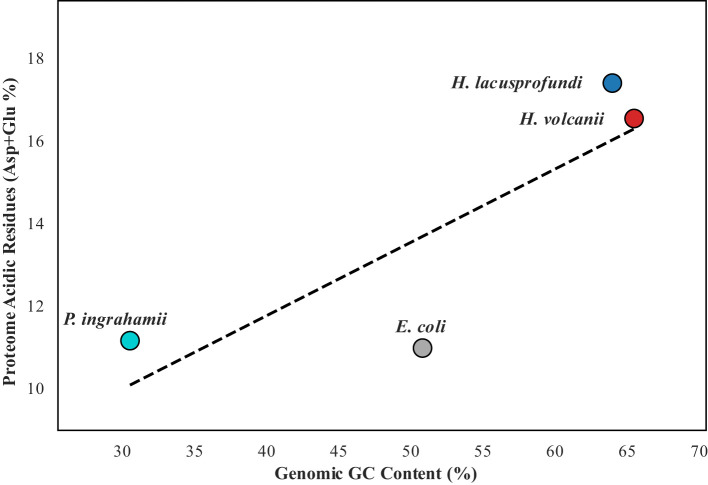
Gene-level relationship between CDS GC content and encoded acidic residue frequency (Asp + Glu %) across four proteomes. Each point represents one protein-coding gene/protein pair. Pearson correlation coefficients were calculated separately for *Escherichia coli, Psychromonas ingrahamii, Haloferax volcanii*, and *Halorubrum lacusprofundi*. Weak correlations across all four datasets indicate that proteome acidification is largely decoupled from GC content.

To determine whether proteome acidification in halophilic and psychrohalophilic systems is a passive consequence of GC-rich genomes or a selectively maintained protein feature, we examined the relationship between CDS GC content and acidic residue frequency of the encoded proteins within each proteome (Figure 7), in the context of earlier proposals linking proteome acidity to genomic nucleotide composition (Kennedy et al., 2001; Soppa, 2006). Pearson correlation analysis across all coding sequences revealed only weak associations in all four organisms: *E. coli* (*r* = 0.059, *p* < 0.001), *H. volcanii* (*r* = −0.049, *p* < 0.01), *P. ingrahamii* (*r* = −0.026, *p* = 0.28), and *H. lacusprofundi* (*r* = 0.059, *p* < 0.05).

These coefficients are all close to zero, indicating that proteins encoded by GC-richer genes are not consistently enriched in acidic residues. Although some correlations reached statistical significance, their effect sizes are negligible and therefore do not support a biologically meaningful dependence of proteome acidification on GC content. Additional cross-proteome comparisons support this conclusion. In the psychrophile *Psychromonas ingrahamii*, a ~20% reduction in genomic GC content relative to *E. coli* did not result in a corresponding decrease in proteomic acidic composition. Despite its AT-biased genome, the baseline acidic residue frequency (~11%) remained comparable to that of the mesophilic control, indicating that nucleotide composition alone does not dictate proteome acidity. Likewise, if proteome acidification were strictly governed by genomic GC content, *Haloferax volcanii* (65.48% GC) would be expected to exhibit greater acidic enrichment than *Halorubrum lacusprofundi* (63.97% GC). However, the opposite pattern was observed, with *H. lacusprofundi* displaying a higher proteome-wide acidic residue content (17.38%) than *H. volcanii* (16.52%).

Together, these results indicate that acidic enrichment is not a passive reflection of nucleotide bias but a selectively maintained structural feature. In *H. volcanii* and especially *H. lacusprofundi*, proteome acidification is therefore more consistent with adaptive selection for protein solubility, hydration stability, and resistance to aggregation under hypersaline and cryo-hypersaline conditions than with passive genomic GC bias alone (Paul et al., 2008). Collectively, these findings support the conclusion that proteome acidification in polyextremophiles is largely decoupled from genomic GC composition and reflects active physicochemical adaptation.

Taken together, the multi-layered analyses support a hierarchical adaptive framework for protein persistence in Antarctic cryo-brines. Here, “hierarchical” refers to adaptation operating at nested organizational scales. Surface electrostatic remodeling and acidic enrichment provide the basal halophilic scaffold that promotes hydration and suppresses aggregation under high ionic strength, consistent with the electrostatic patterns observed across enzymes. At the systems level, proteome-wide acidification and hydropathy redistribution shift the global proteome toward a high-charge, low-hydrophobicity regime favorable for solubility in salt-saturated environments. Superimposed on this background, targeted residue substitutions and selective preservation of conformational flexibility fine-tune functionally important regions required for catalysis at sub-zero temperatures, in agreement with both the substitution analysis in this manuscript and earlier enzyme-level studies on *H. lacusprofundi* (DasSarma et al., 2013; Karan et al., 2020; Laye et al., 2017). The weak relationship between GC content and acidic residue enrichment further indicates that these traits reflect active structural selection rather than passive compositional drift. These converging mechanisms collectively define a molecular framework for psychrohalophilic survival in the Antarctic Deep Lake cryo-brine system and may enhance understanding of adaptation in related cryo-hypersaline environments.

## 4 Conclusion

Comparative structural and proteome-scale analyses indicate that survival of *Halorubrum lacusprofundi* in Antarctic cryo-hypersaline environments is achieved through coordinated molecular remodeling rather than isolated mutations or single adaptive traits. Our findings support a layered adaptive framework in this psychrohalophilic model, in which halophilic solubility determinants provide the basal scaffold, while selective flexibility tuning helps preserve function under low-temperature stress.

Dominant features include extensive surface acidification, reduced hydrophobicity, lowered isoelectric points, targeted residue substitutions, and selective modulation of structural flexibility. Together, these characteristics enhance protein solubility and hydration stability under high ionic strength while preserving catalytic dynamics at sub-zero temperatures.

The integration of surface electrostatic remodeling, proteome-wide hydropathy redistribution, and selective flexibility tuning supports a hierarchical adaptive framework for protein function under combined cold and saline stress. These findings provide mechanistic insight into terrestrial cryo-brine adaptation and contribute to a broader understanding of molecular survival strategies in poly-extreme environments, including saline extraterrestrial systems.

## Data Availability

The original contributions presented in the study are included in the article/Supplementary material, further inquiries can be directed to the corresponding author.
