# Supplementary material for: Coordinated proteome-scale remodeling underlies polyextremophilic survival in Antarctic cryo-hypersaline brines
Source: Front Microbiol. 2026 Apr 17;17:1822442. doi: 10.3389/fmicb.2026.1822442 (PMC13133045; doi:10.3389/fmicb.2026.1822442)
Supplement: Supplementary file 1 [file Supplementary_file_1.docx]

**SUPPLEMENTARY FILE**

**Coordinated Proteome-Scale Remodeling Underlies Polyextremophilic Survival in Antarctic Cryo-Hypersaline Brines**

Shubham Pandey^1#^, Anjali Gupta^1#^, Ashwini Chauhan^1^, Mohammad Ali Amoozegar^2^, Ram Karan^1^*

^1^Department of Microbiology, University of Delhi South Campus, New Delhi, 110021, India

^2^Department of Microbiology, School of Biology, College of Science, University of Tehran, Tehran, 1417864411, Iran

*Corresponding author: R. Karan ([ramkaran@south.du.ac.in](mailto:ramkaran@south.du.ac.in))

#Equally contributing

**Table of Contents**

| **Item** | **Title / Description** | **Page No.** |
| --- | --- | --- |
| Table S1 | Representative enzymes and corresponding UniProt accession numbers used for comparative structural analysis. | 2 |
| Table S2 | **Physicochemical signatures of representative enzymes across environmental gradients.** | 3-4 |
| Table S3 | Proteomic amino acid composition of organisms. | 4 |
| Table S4 | Pairwise amino acid substitution frequencies in *Escherichia. coli, Psychromonas ingrihamii* and *Haloferax volcanii* against *Halorubrum lacusprofundi*. | 5-6 |

**Supplementary Table S1:** Representative enzymes and corresponding UniProt accession numbers used for comparative structural analysis.

| **Enzyme / Protein Family** | ***E. coli***  **(Mesophile)** | ***H. volcanii***  **(Halophile)** | ***P. ingrahamii***  **(Psychrophile)** | ***H. lacusprofundi***  **(Psychrohalophile)** |
| --- | --- | --- | --- | --- |
| Adenylate kinase (Adk) | P69441 | A0A847TUI2 | A1STI3 | B9LQ52 |
| β-galactosidase (β-Gal) | P00722 | A0A6C0UTG9 | A1SWB8 | B9LW38 |
| DNA ligase (LigA) | P15042 | D4GY98 | A1SX61 | B9LU22 |
| Glycerol kinase (GlpK) | P0A6F3 | D4GYI5 | A1SZE1 | B9LMX8 |
| Histidinol-phosphate aminotransferase (HisC) | P06986 | P17736 | A1SVD3 | B9LNJ8 |
| Malate dehydrogenase (MDH) | P61889 | Q9P9L2 | A1SRP8 | B9LU26 |
| Signal recognition particle (SRP54) | P0AGD7 | Q977V2 | A1SZY9 | B9LT33 |

**Supplementary Table S2.** **Physicochemical signatures of representative enzymes across environmental gradients.**

| **Organism** | **Length (AA)** | **MW (kDa)** | **pI** | **Acidic (%)** | **R/K Ratio** | **Aliphatic Index** | **GRAVY** | **H-Bonds per 100 Residues** | **Helix %** | **Strand %** | **Loop %** | **Surface (Exposed) %** | **Core (Buried) %** | **Total Salt Bridges** | **Cavity (A^3^)** |
| --- | --- | --- | --- | --- | --- | --- | --- | --- | --- | --- | --- | --- | --- | --- | --- |
| **Adenylate kinase (Adk)** | | | | | | | | | | | | | | | |
| *E. coli* | 214 | 23.59 | 5.55 | 16.36 | 0.72 | 89.30 | -0.38 | 187.38 | 47.7 | 17.3 | 35.0 | 63.6 | 36.4 | 4 | 1807.94 |
| *P. ingrahamii* | 214 | 23.26 | 4.99 | 16.36 | 0.73 | 86.64 | -0.29 | 180.37 | 46.7 | 13.6 | 39.7 | 62.6 | 37.4 | 4 | 1859.84 |
| *H. volcanii* | 208 | 23.11 | 4.13 | 24.52 | 2.00 | 83.80 | -0.51 | 185.58 | 42.3 | 9.6 | 48.1 | 66.8 | 33.2 | 6 | 1452.48 |
| *H. lacusprofundi* | 207 | 22.97 | 4.05 | 26.57 | 3.25 | 83.33 | -0.47 | 165.22 | 42.0 | 14.0 | 44.0 | 70.0 | 30.0 | 6 | 1564.35 |
| **β-galactosidase (β-Gal)** | | | | | | | | | | | | | | | |
| *E. coli* | 1024 | 116.48 | 5.28 | 12.30 | 3.30 | 77.06 | -0.43 | 133.59 | 9.6 | 27.4 | 63.0 | 51.0 | 49.0 | 47 | 878.65 |
| *P. ingrahamii* | 1035 | 117.46 | 5.23 | 12.85 | 0.91 | 85.15 | -0.36 | 129.86 | 9.7 | 25.2 | 65.1 | 49.4 | 50.6 | 45 | 1000.69 |
| *H. volcanii* | 663 | 74.46 | 4.55 | 16.44 | 7.00 | 66.40 | -0.52 | 184.95 | 54.2 | 6.9 | 38.9 | 61.7 | 38.3 | 21 | 721.66 |
| *H. lacusprofundi* | 700 | 78.06 | 4.40 | 18.57 | 10.8 | 67.63 | -0.55 | 169.86 | 30.0 | 12.7 | 57.3 | 50.6 | 49.4 | 46 | 836.37 |
| **DNA ligase (LigA)** | | | | | | | | | | | | | | | |
| *E. coli* | 671 | 73.61 | 5.39 | 14.75 | 1.44 | 96.63 | -0.14 | 166.92 | 37.4 | 13.1 | 49.5 | 63.8 | 36.2 | 77 | 2632.99 |
| *P. ingrahamii* | 649 | 71.53 | 5.54 | 13.56 | 0.33 | 93.42 | -0.17 | 161.02 | 34.2 | 10.6 | 55.2 | 63.9 | 36.1 | 16 | 2360.89 |
| *H. volcanii* | 699 | 75.81 | 4.35 | 20.74 | 6.20 | 84.01 | -0.36 | 166.09 | 36.8 | 16.0 | 47.2 | 66.2 | 33.8 | 79 | 1400.00 |
| *H. lacusprofundi* | 710 | 77.22 | 4.29 | 20.85 | 8.57 | 81.25 | -0.41 | 161.83 | 34.1 | 9.2 | 56.8 | 64.1 | 35.9 | 37 | 1242.88 |
| **Glycerol kinase (GlpK)** | | | | | | | | | | | | | | | |
| *E. coli* | 502 | 56.23 | 5.36 | 13.35 | 1.45 | 84.34 | -0.29 | 157.97 | 32.1 | 13.5 | 54.4 | 50.8 | 49.2 | 21 | 554.49 |
| *P. ingrahamii* | 507 | 55.78 | 5.40 | 12.03 | 0.85 | 86.61 | -0.19 | 168.64 | 31.8 | 14.0 | 54.2 | 51.9 | 48.1 | 22 | 636.11 |
| *H. volcanii* | 510 | 56.74 | 4.36 | 18.63 | 2.58 | 76.57 | -0.49 | 174.51 | 32.5 | 14.5 | 52.9 | 50.8 | 49.2 | 31 | 831.69 |
| *H. lacusprofundi* | 513 | 56.95 | 4.22 | 19.30 | 2.45 | 76.65 | -0.52 | 174.27 | 31.2 | 14.2 | 54.6 | 52.4 | 47.6 | 17 | 542.62 |
| **Histidinol-phosphate aminotransferase (HisC)** | | | | | | | | | | | | | | | |
| *E. coli* | 356 | 39.36 | 5.01 | 11.52 | 1.75 | 101.12 | -0.04 | 162.64 | 38.2 | 9.3 | 52.5 | 53.1 | 46.9 | 11 | 1131.42 |
| *P. ingrahamii* | 356 | 39.07 | 5.30 | 10.96 | 0.83 | 102.84 | 0.09 | 170.51 | 34.3 | 13.8 | 52.0 | 59.0 | 41.0 | 20 | 395.26 |
| *H. volcanii* | 361 | 39.42 | 4.40 | 18.56 | 2.62 | 81.11 | -0.34 | 157.34 | 35.5 | 12.2 | 52.4 | 52.9 | 47.1 | 11 | 1213.00 |
| *H. lacusprofundi* | 364 | 39.33 | 4.43 | 18.68 | 5.00 | 83.68 | -0.33 | 165.66 | 34.1 | 11.3 | 54.7 | 54.7 | 45.3 | 10 | 1311.55 |
| **Malate dehydrogenase (MDH)** | | | | | | | | | | | | | | | |
| *E. coli* | 312 | 32.34 | 5.61 | 10.26 | 0.38 | 105.32 | 0.19 | 180.45 | 44.2 | 7.7 | 48.1 | 54.8 | 45.2 | 8 | 1170.43 |
| *P. ingrahamii* | 319 | 33.00 | 4.94 | 10.66 | 0.33 | 113.17 | 0.35 | 177.43 | 42.3 | 10.3 | 47.3 | 58.3 | 41.7 | 6 | 1331.40 |
| *H. volcanii* | 304 | 32.65 | 4.20 | 19.08 | 1.30 | 87.20 | -0.25 | 172.04 | 41.1 | 10.9 | 48.0 | 56.2 | 43.8 | 19 | 955.07 |
| *H. lacusprofundi* | 304 | 32.64 | 4.23 | 19.41 | 1.67 | 85.30 | -0.31 | 175.66 | 42.1 | 9.5 | 48.4 | 56.9 | 43.1 | 14 | 979.26 |
| **Signal recognition particle 54 (SRP54)** | | | | | | | | | | | | | | | |
| *E. coli* | 453 | 49.79 | 9.52 | 12.80 | 0.65 | 90.40 | -0.24 | 186.09 | 56.7 | 7.3 | 36.0 | 62.9 | 37.1 | 20 | 1005.63 |
| *P. ingrahamii* | 457 | 49.73 | 9.55 | 12.69 | 0.53 | 90.53 | -0.23 | 191.25 | 57.1 | 5.3 | 37.6 | 63.2 | 36.8 | 27 | 1058.20 |
| *H. volcanii* | 465 | 50.92 | 4.35 | 19.78 | 0.96 | 79.27 | -0.48 | 162.75 | 29.1 | 14.6 | 56.3 | 49.3 | 50.7 | 36 | 939.40 |
| *H. lacusprofundi* | 464 | 50.89 | 4.37 | 20.26 | 1.33 | 75.69 | -0.50 | 199.35 | 56.0 | 5.8 | 38.1 | 58.0 | 42.0 | 19 | 1115.93 |

**Supplementary Table S3.** Proteomic amino acid composition of organisms.

| **Organism** | **Total Residues Analyzed** | **Small (Ala+Gly) %** | **Aromatic (Phe+Tyr+Trp) %** |
| --- | --- | --- | --- |
| *E. coli* (Mesophile) | 1,354,434 | 16.87 | 8.27 |
| *P. ingrahamii* (Psychrophile) | 1,173,915 | 14.87 | 8.61 |
| *H. volcanii* (Halophile) | 1,115,492 | 19.63 | 7.29 |
| *H. lacusprofundi* (Polyextremophile) | 1,011,093 | 19.68 | 7.03 |

**Supplementary Table S4:** Pairwise amino acid substitution frequencies in *E. coli, Psychromonas ingrihamii* and *Haloferax volcanii* against *H. lacusprofundi*.

|  | **Amino acid substitutions in *E. coli* vs *H. lacusprofundi*** | | | | | | | | | | | | | | | | | | | | |
| --- | --- | --- | --- | --- | --- | --- | --- | --- | --- | --- | --- | --- | --- | --- | --- | --- | --- | --- | --- | --- | --- |
|  | ***H. lacusprofundi*** | | | | | | | | | | | | | | | | | | | | |
|  |  | A | C | D | E | F | G | H | I | K | L | M | N | P | Q | R | S | T | V | W | Y |
| ***E. coli*** | A | 0 | 0 | 29 | 29 | 3 | 21 | 6 | 7 | 2 | 20 | 4 | 9 | 13 | 14 | 28 | 14 | 22 | 26 | 2 | 7 |
|  | C | 2 | 0 | 5 | 4 | 0 | 3 | 2 | 1 | 1 | 3 | 0 | 0 | 2 | 1 | 5 | 3 | 0 | 5 | 0 | 0 |
|  | D | 22 | 4 | 0 | 18 | 4 | 17 | 1 | 7 | 3 | 12 | 3 | 1 | 12 | 5 | 13 | 10 | 11 | 12 | 1 | 6 |
|  | E | 23 | 2 | 23 | 0 | 6 | 13 | 4 | 12 | 3 | 28 | 3 | 8 | 12 | 4 | 12 | 13 | 15 | 19 | 7 | 5 |
|  | F | 10 | 1 | 15 | 8 | 0 | 8 | 4 | 7 | 2 | 6 | 4 | 3 | 5 | 1 | 7 | 3 | 5 | 10 | 0 | 3 |
|  | G | 29 | 3 | 27 | 24 | 10 | 0 | 3 | 4 | 10 | 14 | 5 | 7 | 9 | 9 | 14 | 4 | 14 | 25 | 4 | 6 |
|  | H | 7 | 1 | 6 | 9 | 1 | 5 | 0 | 5 | 0 | 0 | 1 | 0 | 3 | 3 | 1 | 3 | 4 | 4 | 0 | 4 |
|  | I | 24 | 1 | 5 | 17 | 9 | 14 | 3 | 0 | 4 | 13 | 3 | 5 | 8 | 2 | 8 | 7 | 7 | 12 | 6 | 6 |
|  | K | 22 | 0 | 11 | 16 | 4 | 12 | 1 | 11 | 0 | 7 | 2 | 2 | 9 | 8 | 7 | 6 | 9 | 20 | 2 | 8 |
|  | L | 34 | 2 | 34 | 21 | 10 | 27 | 9 | 10 | 8 | 0 | 5 | 7 | 14 | 9 | 17 | 14 | 14 | 22 | 5 | 6 |
|  | M | 7 | 0 | 4 | 7 | 7 | 6 | 2 | 4 | 4 | 7 | 0 | 1 | 7 | 4 | 1 | 1 | 4 | 2 | 2 | 1 |
|  | N | 8 | 3 | 10 | 12 | 2 | 11 | 4 | 2 | 0 | 15 | 2 | 0 | 9 | 3 | 12 | 9 | 7 | 6 | 3 | 2 |
|  | P | 13 | 2 | 24 | 14 | 6 | 13 | 5 | 10 | 3 | 6 | 0 | 8 | 0 | 2 | 7 | 6 | 5 | 12 | 3 | 7 |
|  | Q | 14 | 0 | 20 | 13 | 1 | 14 | 2 | 2 | 3 | 7 | 5 | 1 | 9 | 0 | 9 | 4 | 9 | 11 | 1 | 5 |
|  | R | 14 | 2 | 24 | 14 | 5 | 20 | 3 | 7 | 3 | 17 | 3 | 4 | 7 | 7 | 0 | 13 | 13 | 12 | 1 | 9 |
|  | S | 9 | 3 | 18 | 10 | 9 | 11 | 3 | 1 | 2 | 10 | 5 | 5 | 8 | 5 | 12 | 0 | 12 | 13 | 2 | 3 |
|  | T | 24 | 1 | 15 | 25 | 4 | 7 | 3 | 2 | 5 | 16 | 2 | 6 | 6 | 3 | 11 | 9 | 0 | 19 | 2 | 3 |
|  | V | 25 | 2 | 29 | 20 | 7 | 22 | 4 | 9 | 5 | 24 | 3 | 5 | 17 | 14 | 26 | 18 | 13 | 0 | 4 | 5 |
|  | W | 6 | 0 | 6 | 5 | 2 | 2 | 1 | 2 | 0 | 3 | 0 | 2 | 2 | 0 | 4 | 2 | 1 | 4 | 0 | 2 |
|  | Y | 9 | 0 | 9 | 8 | 2 | 8 | 2 | 2 | 0 | 4 | 0 | 3 | 3 | 0 | 7 | 3 | 8 | 6 | 0 | 0 |
|  | **Amino acid substitutions in *P. ingrahamii* vs *H. lacusprofundi*** | | | | | | | | | | | | | | | | | | | | |
|  | ***H. lacusprofundi*** | | | | | | | | | | | | | | | | | | | | |
|  |  | A | C | D | E | F | G | H | I | K | L | M | N | P | Q | R | S | T | V | W | Y |
| ***P. ingrahamii*** | A | 0 | 4 | 35 | 27 | 7 | 25 | 4 | 7 | 6 | 19 | 5 | 2 | 18 | 13 | 21 | 13 | 17 | 25 | 3 | 7 |
|  | C | 3 | 0 | 3 | 7 | 0 | 6 | 0 | 0 | 1 | 1 | 0 | 2 | 0 | 1 | 5 | 2 | 0 | 1 | 0 | 3 |
|  | D | 13 | 3 | 0 | 32 | 6 | 17 | 2 | 5 | 4 | 13 | 4 | 3 | 14 | 9 | 11 | 5 | 11 | 15 | 5 | 6 |
|  | E | 25 | 1 | 22 | 0 | 4 | 17 | 4 | 11 | 4 | 22 | 2 | 6 | 10 | 4 | 14 | 8 | 7 | 14 | 4 | 5 |
|  | F | 17 | 0 | 11 | 8 | 0 | 15 | 2 | 4 | 2 | 5 | 1 | 6 | 4 | 4 | 8 | 4 | 8 | 7 | 0 | 1 |
|  | G | 24 | 2 | 20 | 22 | 7 | 0 | 5 | 6 | 5 | 15 | 5 | 5 | 10 | 7 | 11 | 10 | 17 | 24 | 3 | 9 |
|  | H | 7 | 1 | 10 | 4 | 1 | 3 | 0 | 5 | 1 | 5 | 1 | 2 | 6 | 2 | 2 | 3 | 3 | 4 | 0 | 3 |
|  | I | 23 | 1 | 24 | 19 | 7 | 17 | 2 | 0 | 6 | 16 | 2 | 7 | 10 | 3 | 15 | 11 | 11 | 17 | 3 | 4 |
|  | K | 20 | 2 | 16 | 19 | 4 | 22 | 4 | 13 | 0 | 17 | 5 | 4 | 8 | 7 | 15 | 9 | 13 | 18 | 5 | 6 |
|  | L | 33 | 2 | 26 | 29 | 8 | 23 | 5 | 11 | 6 | 0 | 4 | 7 | 13 | 6 | 14 | 17 | 11 | 25 | 5 | 12 |
|  | M | 7 | 0 | 10 | 4 | 4 | 8 | 0 | 4 | 4 | 4 | 0 | 1 | 6 | 4 | 6 | 3 | 3 | 3 | 1 | 1 |
|  | N | 13 | 2 | 12 | 14 | 4 | 9 | 2 | 5 | 0 | 12 | 5 | 0 | 5 | 3 | 4 | 5 | 12 | 15 | 1 | 3 |
|  | P | 9 | 0 | 17 | 10 | 4 | 10 | 5 | 4 | 1 | 13 | 2 | 5 | 0 | 4 | 10 | 7 | 7 | 10 | 1 | 1 |
|  | Q | 15 | 0 | 15 | 13 | 7 | 10 | 1 | 4 | 4 | 8 | 4 | 1 | 10 | 0 | 11 | 5 | 5 | 14 | 3 | 3 |
|  | R | 14 | 2 | 8 | 6 | 2 | 8 | 5 | 8 | 1 | 9 | 5 | 2 | 5 | 2 | 0 | 7 | 13 | 10 | 2 | 5 |
|  | S | 17 | 2 | 15 | 17 | 12 | 14 | 8 | 5 | 3 | 19 | 1 | 3 | 11 | 5 | 14 | 0 | 11 | 12 | 5 | 6 |
|  | T | 17 | 0 | 21 | 16 | 3 | 10 | 5 | 4 | 7 | 16 | 2 | 6 | 6 | 9 | 18 | 8 | 0 | 14 | 5 | 1 |
|  | V | 29 | 1 | 26 | 21 | 7 | 17 | 5 | 7 | 2 | 19 | 6 | 5 | 12 | 8 | 17 | 14 | 13 | 0 | 0 | 10 |
|  | W | 4 | 2 | 8 | 4 | 1 | 1 | 1 | 1 | 0 | 0 | 0 | 0 | 4 | 0 | 2 | 1 | 5 | 3 | 0 | 2 |
|  | Y | 10 | 2 | 11 | 9 | 3 | 8 | 1 | 2 | 2 | 3 | 1 | 6 | 4 | 3 | 6 | 1 | 5 | 7 | 1 | 0 |
|  | **Amino acid substitutions in *H. volcanii* vs *H. lacusprofundi*** | | | | | | | | | | | | | | | | | | | | |
|  | ***H. lacusprofundi*** | | | | | | | | | | | | | | | | | | | | |
|  |  | A | C | D | E | F | G | H | I | K | L | M | N | P | Q | R | S | T | V | W | Y |
| ***H. volcanii*** | A | 0 | 0 | 23 | 26 | 8 | 26 | 6 | 6 | 4 | 10 | 1 | 4 | 11 | 6 | 11 | 15 | 11 | 16 | 4 | 4 |
|  | C | 2 | 0 | 1 | 1 | 0 | 1 | 1 | 0 | 0 | 1 | 0 | 1 | 1 | 0 | 3 | 0 | 1 | 2 | 0 | 1 |
|  | D | 19 | 0 | 0 | 39 | 5 | 18 | 3 | 8 | 1 | 16 | 1 | 9 | 8 | 5 | 19 | 11 | 11 | 10 | 3 | 5 |
|  | E | 33 | 2 | 37 | 0 | 3 | 10 | 1 | 7 | 5 | 19 | 2 | 2 | 7 | 3 | 8 | 6 | 9 | 16 | 3 | 4 |
|  | F | 5 | 1 | 8 | 7 | 0 | 4 | 3 | 1 | 0 | 2 | 2 | 1 | 5 | 1 | 2 | 3 | 3 | 4 | 0 | 1 |
|  | G | 14 | 2 | 11 | 16 | 1 | 0 | 4 | 6 | 4 | 9 | 2 | 1 | 10 | 3 | 13 | 5 | 14 | 15 | 2 | 3 |
|  | H | 4 | 0 | 3 | 5 | 0 | 3 | 0 | 1 | 0 | 4 | 0 | 1 | 1 | 1 | 3 | 1 | 3 | 2 | 0 | 1 |
|  | I | 3 | 1 | 7 | 3 | 1 | 7 | 1 | 0 | 0 | 4 | 3 | 4 | 3 | 2 | 2 | 1 | 2 | 10 | 3 | 1 |
|  | K | 5 | 0 | 6 | 4 | 0 | 3 | 1 | 0 | 0 | 3 | 1 | 3 | 1 | 5 | 5 | 2 | 4 | 3 | 0 | 0 |
|  | L | 17 | 1 | 15 | 16 | 4 | 14 | 0 | 2 | 0 | 0 | 5 | 7 | 7 | 7 | 10 | 6 | 9 | 16 | 2 | 4 |
|  | M | 2 | 0 | 1 | 0 | 1 | 2 | 0 | 1 | 0 | 6 | 0 | 1 | 0 | 1 | 2 | 1 | 3 | 2 | 1 | 1 |
|  | N | 6 | 0 | 5 | 3 | 2 | 7 | 1 | 4 | 1 | 3 | 1 | 0 | 5 | 4 | 4 | 6 | 1 | 2 | 0 | 3 |
|  | P | 8 | 1 | 12 | 7 | 3 | 5 | 3 | 2 | 3 | 5 | 1 | 1 | 0 | 6 | 3 | 5 | 4 | 8 | 3 | 2 |
|  | Q | 10 | 1 | 7 | 6 | 0 | 6 | 1 | 1 | 3 | 2 | 0 | 0 | 5 | 0 | 8 | 3 | 2 | 4 | 0 | 1 |
|  | R | 12 | 1 | 14 | 16 | 8 | 5 | 2 | 1 | 4 | 10 | 4 | 4 | 8 | 1 | 0 | 7 | 8 | 9 | 3 | 7 |
|  | S | 16 | 0 | 16 | 9 | 3 | 6 | 4 | 4 | 1 | 3 | 0 | 2 | 7 | 1 | 8 | 0 | 13 | 10 | 3 | 3 |
|  | T | 23 | 0 | 17 | 7 | 4 | 9 | 0 | 7 | 1 | 12 | 0 | 4 | 7 | 3 | 11 | 8 | 0 | 9 | 0 | 4 |
|  | V | 23 | 1 | 12 | 13 | 4 | 11 | 2 | 8 | 2 | 16 | 3 | 4 | 8 | 7 | 16 | 9 | 12 | 0 | 1 | 4 |
|  | W | 2 | 0 | 6 | 5 | 1 | 1 | 2 | 2 | 0 | 3 | 0 | 2 | 0 | 0 | 2 | 0 | 1 | 3 | 0 | 1 |
|  | Y | 7 | 0 | 4 | 2 | 3 | 4 | 3 | 0 | 1 | 6 | 0 | 1 | 3 | 3 | 2 | 3 | 2 | 5 | 1 | 0 |
